# Emerging frontiers in juice processing: The role of ultrasonication and other non-thermal technologies in enhancing antioxidant capacity and quality of fruit and vegetable juices

**DOI:** 10.1016/j.ultsonch.2025.107554

**Published:** 2025-09-09

**Authors:** Muhammad Umair, Muhammad Abid, Mishal Mumraiz, Saqib Jabbar, Song Xun, Kashif Ameer, Muhammad Shahid Riaz Rajoka, He Zhendan, Saqer S. Alotaibi, Robert Mugabi, Gulzar Ahmad Nayik

**Affiliations:** a College of Pharmacy, Shenzhen Technology University, 518118 Shenzhen, China; b Institute of Food Science & Nutrition, University of Sargodha, Sargodha 40100, Pakistan; c National Institute of Food Science and Technology, University of Agriculture Faisalabad, Pakistan; d Food Science Research Institute (FSRI), National Agricultural Research Centre (NARC), Islamabad, Pakistan; e Department of Food Science and Technology, College of Chemistry and Environmental Engineering, Shenzhen University, Shenzhen, China; f Department of Biotechnology, College of Science, Taif University, P.O.Box 11099, Taif 21944, Saudi Arabia; g Department of Food Technology and Nutrition, Makerere University, Kampala, Uganda; h Marwadi University Research Centre, Department of Microbiology, Marwadi University, Rajkot 360003 Gujarat, India

**Keywords:** Nonthermal treatment, Ultrasonication, Cold plasma, Irradiation, Juice quality, Antioxidant capacity

## Abstract

Fruit and vegetable juices (FVJs) are widely consumed due to their rich profile of vitamins, minerals, and bioactive compounds, particularly antioxidants, which play a critical role in mitigating non-communicable diseases. However, conventional thermal processing methods, while effective in microbial inactivation, significantly compromise the antioxidant capacity (AC), sensory attributes, and nutritional quality of FVJs. In response to growing consumer demand for minimally processed and functionally enriched juices, non-thermal technologies (NTTs) have emerged as promising alternatives. This review comprehensively evaluates recent advancements (2015–2024) in non-thermal processing methods—including ultrasonication, cold plasma, irradiation, and pulsed electric fields—and their influence on the AC and overall quality of FVJs.

NTTs enhance juice quality by inducing abiotic stress, activating antioxidant biosynthesis pathways, inactivating spoilage enzymes, and facilitating the release of bound phytochemicals. The review highlights the mechanistic insights, optimal processing parameters, and synergistic effects of combined NTT approaches on juice matrix, antioxidant retention, and enzymatic stability. Furthermore, it discusses the scalability, energy efficiency, and environmental sustainability of these technologies in comparison to traditional thermal methods. Despite promising outcomes at the laboratory scale, commercial adaptation of NTTs faces challenges such as equipment cost, process optimization for diverse juice matrices, regulatory acceptance, and consumer perception. Future directions emphasize the need for multidisciplinary collaborations to decode antioxidant metabolism, validate safety parameters (e.g., ROS/RNS residues), and develop industry-ready, cost-effective, and safe processing units. This review stands out by providing mechanistic insights, comparative efficacy, and optimization strategies of various NTTs specifically aimed at enhancing antioxidant capacity in fruit and vegetable juices. It also integrates a critical discussion on industrial scalability, regulatory challenges, and future biosynthetic pathway investigations, distinguishing it from prior reviews.

## 1 Introduction

Organic fluid extracts of different cells and tissues obtained from vegetables and ripened fruits by a mechanical method without the use of a heat source or any other liquid medium as a solvent are regarded as fruit and vegetable juice (FVJs)[1,2]. FVJs are a natural, valuable, and vital part of human balanced nutrition and are frequently purchased by consumers in order to meet their daily nutrient requirements and promote good health in a fast and convenient manner[3]. The National Health Service (UK) claims that 150 ml of 100 % FVJs can effectively replace the everyday requirement of five portions of fruits and vegetables (“5 a day” recommendation) in a day[4]. Scientists observed that compounds that are biologically active in the human body are considerably easier to digest or assimilate when consumed through FVJs as compared to the entire tissue of the fruit or vegetable[5].

Natural antioxidants present in FVJs could be associated with the reduced risk of metabolic disorders and they can improve the immune system, thereby promoting human health, and that’s why FVJs are a popular beverage choice among consumers[6]. The Antioxidant capacity (AC) of FVJs is largely provided by vitamin A (carotenoids, lycopene), vitamin C, and polyphenol content, apart from vitamin E[7]. However, according to the FDA, FVJs have a low acidity (pH between 5 and 6), which are categorized as potentially unsafe[8]. And it is crucial to treat the FVJs to prevent microbial spoilage. The capability of commercially available traditional thermal treatments of FVJs in controlling microbial spoilage is good enough but also damages their phytochemicals, colour, flavour, and diminishes nutritional value due to the breakdown of proteins, vitamins, and aromatic sensory components, thus adversely affecting the AC of FVJs[7]. The above-mentioned challenges have inspired the researchers to discover novel and innovative technologies for preserving the nutritional quality and AC of FVJs with prolonged shelf life.

Recent innovations in food processing address consumer concerns about chemical additives and thermal processing. Non-thermal technologies (NTTs) offer promising benefits, including enhanced food safety, extended shelf-life, and minimal nutritional damage (Wang et al. 2022, [9,10,7]. The word “nonthermal” refers to processes that can inactivate microorganisms without the application of extensive-heat[11]. Non-thermal technologies (NTTs) like ultrasonication, irradiation, pulse electric field, and high voltage cold plasma offer several benefits. These include enhanced shelf-life by inhibiting spoilage-causing enzymes, retention of bioactive compounds and flavor/texture constituents. NTTs also improved energy efficiency, higher nutritional quality compared to thermal processing, particularly in fruit and vegetable juices (FVJs)[12,5,11]. It is reported that NTTs can induce abiotic stress on fruit or vegetable tissues, activate the antioxidant system, accumulate ROS species, and cause the movement of carbohydrates and starch to sink. All this together can cause damaging effects in fruit and vegetable cells at the tissue level and thus increase AC[13,7]. In addition, the impact of NTTs on the antioxidant system, reactive species, and AC is unclear and needs to be addressed.

Therefore, NTTs are good substitutes for conventional heat processing since they help preserve the characteristics of the original FVJs in the treated juice sample. While previous reviews have broadly discussed non-thermal technologies for food processing, few have critically and comprehensively evaluated their mechanistic impacts, process optimization, and potential to enhance antioxidant capacity specifically in fruit and vegetable juices. Our review aims to fill this gap by not only summarizing the advances in ultrasonication, irradiation, pulsed electric fields, and cold plasma, but also highlighting their comparative effectiveness, challenges in scale-up, and directions for future exploration. This focused and integrative approach offers new insights for researchers and industry stakeholders working at the interface of juice quality enhancement and sustainable processing.

### 1.1 Processed fruit and vegetable juices health benefits

Fruit and vegetable juices (FVJs) have been considered very nutritionally rich, and could play a health promoting factor. Processed FVJs are important healthy foods owing to their convenient nature as a source of essential nutrients, including vitamins (such as vitamin C, folate, and provitamin A), minerals (like potassium and magnesium), phytochemicals (e.g., flavonoids, carotenoid, and phenolic acids), and dietary antioxidants in prevention and functional health. The rising number of consumers demanding juice products with an improved bioactive profile has prompted advancement in the processing technologies to maintain or even amplify the health benefit of the products[14].

Daily intake of processed FVJs has been related to many physiological advantages. These are enhanced cardiovascular health state, decreased oxidative stress, improved immune system, and gastrointestinal health. Most fruit and vegetable matrices contain polyphenols and flavonoids that prove to be potent antioxidants and inflammation inhibitors, thereby assisting in reducing chronic disorders including atherosclerosis, diabetes type 2, certain malignancies, and neurodegenerative disorders. An example is juices of pomegranates, blueberries and beetroot that have enormous potentials in lowering blood pressure and enhancing vascular activity due to the abundant polyphenolic and nitrate content present in the juices[15].

Widespread thermal processes that are used to achieve microbial safety and shelf-life of juices may also have adverse effects on natural vitamins and phytochemicals in juices by destroying thermo-sensitive ones. Non-thermal processing technologies, on the other hand, which include ultra sonication, pulsed electric field (PEF), high pressure processing (HPP) and cold plasma are also intentioned on preserving and enriching the nutritional and sensorial quality of juices with minimal nutrient losses. In specific, ultrasonication can be used to disrupt cell walls so that free and bound phenolics can be released and extracted with greater ease, which ultimately increases antioxidant potential of the juice[16].

Recent findings indicate that ultrasonication has the ability to considerably enhance total phenolic content (TPC), total flavonoid content (TFC), and radical scavenging activity (DPPH, ABTS) of different juices that include carrot, orange, grape, and tomato juice. Not only does this increase in antioxidant properties increase juice stability and shelf-life but they also translate to the possibility of bringing about a reduction in oxidative stress when consumed. Also, biological activity of processed juices can be attributed to the preservation of natural enzymes such as superoxide dismutase (SOD) and catalase which are usually destroyed during thermal treatments. Moreover, some minimally clarified juice contains dietary fiber fraction, which is beneficial to digestive health, and it can adjust glycemic reactions. Non-thermal procedures enable the fractions of insoluble and soluble fibers that would be decreased in the typical processing to be kept whole. Processed vegetable juice products like kale, beetroot juice, and celery juice have been associated with liver detoxification, electrolyte reduction, and fatigue reduction processes, in particular, because they retain micronutrients and bioactive compounds[17].

## 2 Ultrasonication

Ultrasonication (US) could be employed as a substitute for traditional thermal pasteurization for FVJs[18]. Currently, US is a relatively new non-thermal technology in the FVJs industry. According to the basic term, a sound wave with a frequency greater than 20 kHz, (greater than the average of human perception), is known as “ultrasound”[19]. In the US process, a variety of expansion and compression effects are caused by ultrasonic waves when these waves pass through a medium. The presence of air causes the creation of tiny cavities along the chambers, which induces the expansion of these cavities to their maximum size before collapsing. Where the walls of these apertures breakdown and release a substantial amount of energy, resulting in increasing heat and mass transfer rates[20]. While, due to pressure fluctuation, the dispersion of ultrasonic waves in a fluid causes bubbles to rupture, these generate tiny bubbles when they breakdown, causing a rise in the normal temperature as well as pressure in FVJs. As a result, the high pressure and intensive local energy produced without creating a considerable rise in temperature had a selective pasteurization or sterilization effect in FVJs[21,22].

### 2.1 Types of ultrasonication

**Fig. 1:**
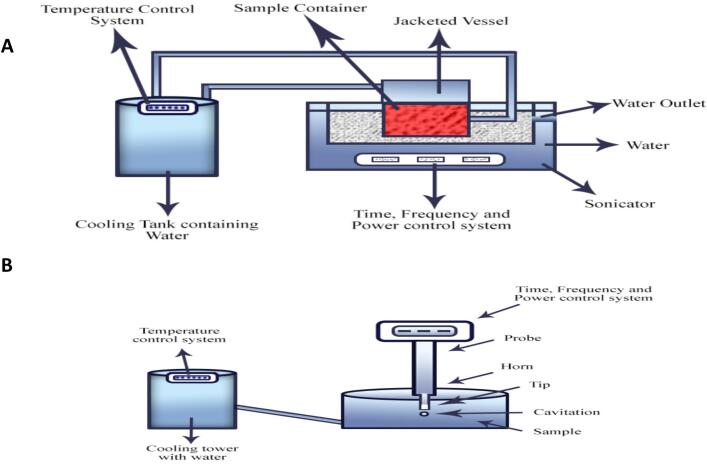
Schematic diagram of sonication systems with (A) sonication water bath and (B) sonication with probe configurations, adapted from[25]with permission.

The US is applied with diverse frequencies and is categorized as: 1) high-frequency ultrasonication (1 MHz–100 MHz); 2) medium-frequency ultrasonication (100 kHz − 1 MHz); 3) low-frequency ultrasonication (20 kHz–100 kHz). The US with less energy (less power, low intensity) had a frequency higher than 100 kHz and an intensity less than 1 W/cm^2^. Whereas, US with higher energy (more power, high intensity) employs intensity greater than 1 W/cm^2^ at frequencies ranging from 20 to 500 kHz [23]. The US is performed with the use of an ultrasonic device inserted in a fluid or juice mixture and processed at a specific frequency called a sonotrode, whereas the US can be accomplished when a food product is in packed form and placed in an ultrasonic water bath. A bath generates sound waves that produce an ultrasound effect and cause the required alternations in a variety of food items[24]. The schematic diagram of US systems with a water bath (ultrasonic water bath) and probe configurations (sonotrodes) used in FVJs is shown in Fig. 1 **(A-B)**. It is worth mentioning that low-frequency ultrasonication generates more forces than high-frequency ultrasonication; however, in the case of medium-frequency ultrasonication, the forces (chemical radicals) are parallel to a surface or cross section of a body; hence, it is thought to be an ideal frequency range for FVJs processing, facilitated by sonochemistry. However, the creation of free radicals may cause adverse effects on FVJs[19].

The US used in the FVJs preparation, evaluation, and quality assurance is mainly based on various process parameters such as ultrasonication frequency (UF), ultrasonication power (UP), and ultrasonication time (UT)[26]. The impact of each US parameter on the AC will be reviewed and summarized in the following sections: The impact of UF on the AC was observed, and it was found that the highest AC was observed when the UF was set to single-frequency (35 kHz) compared to double- or triple-frequency. Whereas, UP at 120 W was found to be highest in the AC when compared with other powers (30, 60, 150, and 180 W), indicating that excessive ultrasonication could go against further increasing the AC. Other studies also critically reviewed the UP effect on the food matrix of various FVJs and found that UP can lead to higher cell disruption and ultimately increase the extraction capacity of bioactive compounds that possess antioxidant properties and may cause the enhancement of AC[27].

However, in the case of UT, it is noted that UT positively affects the AC to some extent, and then the AC starts to decrease[28]. Some scholars have noticed that high UT and high UP may cause an opposite or negative effect on AC[1], indicating that UT plays a critical role for AC, as too high UT in FVJs might lead to the damage of active sites, whereas when the UT is too short, it may not be able to fully expose the antioxidant active sites[26].It should be noted that such effects are caused by “choked cavitation”[29]. It is also reported that AC was decreased during storage, and this might be due to the lower stability of phenolic compounds that have antioxidant properties[20]. As antioxidant properties have a correlation with phenolic contents, which decompose after storage for a longer period, some studies have shown better extraction (phenolic compounds) and inactivation of enzymes (spoilage-causing) due to US, which also results in the enhancement of AC[30]. In addition, structural changes induced by the US also play an important role in AC. For example, pectin contents in FVJs with lower molecular weight and higher α-D-1, 4-galactronic acid (GalA) contents showed better AC due to exposure to a more active (antioxidant) site, could produce more reducing ends, and possessed higher reducing capacity[26]. Therein, UT moderately positively impacts AC, with high UT and high UP potentially having opposite effects. UT is critical for antioxidant activity, as excessively high UT can damage active sites and short UT may not fully expose antioxidant sites.

### 2.2 Mechanism of US process parameters on AC and other quality attributes in FVJs

The possible reason behind the enhancement of AC by US could be due to UV waves in the liquid (due to the formation of unstable bubbles), which collapse with each other and produce shockwaves. These shockwaves rotate in a sonic field, cause micro-streaming and turbulence, and result in a stable, small-scale cavitational effect. The mechanism of the power ultrasound is depicted in Fig. 9.

However, when US treatment is prolonged, in the second phase, transient cavitation may occur due to the rapid formation of large bubbles within a short time, resulting in a large amount of pressure and stress. Altogether, these two phases of cavitational effect enhance the free hydroxyl radical generation, and adding a second hydroxyl group in the ortho or *para*-positions of phenolics thus increases AC[20,31]. When fruit juices are subjected to US, cell membranes are punctured (thinning), free radicals are produced, and the intracellular matrix is released. The strength and form of the treatment determine the characteristics of the US-induced waves. Air bubbles and pressure are created by sound waves. The shift in pressure, as well as the depression and contraction of medium-sized molecules, causes rupturing in the cell walls and the release of bound forms of antioxidant compounds. There are two forms of cavitation phenomena (acoustic cavitation): transient and stable cavitation, both of which generate shock waves and gas bubbles due to the improper air vibrations[25]. Additionally, free hydroxyl radicals produced in the US can attach to the aromatic ring of phenolic compounds[32]. Additionally, US-induced cavitation enhanced tissue decomposition and interaction between pulp components and particles, thus enhancing the overall AC[32]. US-induced waves, created by sound waves, cause air bubbles and pressure shifts, causing cell wall rupturing and antioxidant compound release.US-induced cavitation enhances tissue decomposition and interaction between pulp components and particles, improving overall AC. These factors could potentially contribute to the enhancement of AC in FVJs. In addition, the key findings of various US process variables on FVJs quality parameters have been listed in Table 1. However, further study efforts encompassing rheological aspects and other desired characteristics would increase the effectiveness and reduce the time consumed in the non-destructive and rapid assessment of AC in FVJs.

**Table 1:** Key findings of various ultrasonication variables on AC and other quality parameters in FVJs.

**Source(s) of Fruit**	**Product**	**Conditions for Processing**	**Result(s)**	**Reference**
Barberry	Juice	Sonication power: 200 W; Amplitude: 70 % & 100 %; Frequency: 20 kHz; Sonication time: 15 min; Temperature: 25 °C	US at 70 % power has improved barberry juice quality, microbial count ↓ total phenolic contents (TPC) ↑ and AC↑. No effect on anthocyanin and juice color	[33]
Carrot	Juice	Frequency: 20 kHz, Amplitude: 80 %, Time: 3 min; Intensity: 46 Wcm^2^; Temperature: 10 °C	The US treatment has positive effect on carrot juice, microbial count↓ and sugar contents (sucrose, fructose, and glucose)↓whereas, chlorogenic acid, total carotenoid, lycopene, and lutein contents and AC↑	[34]
Strawberry	Juice	Frequency: 40 kHz; Sonication power: 180 W; Time: 0, 15, 30 min. Time duration: 14 days; Temperature; 5 °C	Inoculated pathogens ↓, native microorganisms (probiotic) were stable, where no effect on bioactive compounds and AC.	[35]
Cloudy apple	Juice	Frequency: 30 kHz; Sonication power: 100 W (probes with 10 and 20 mm tip diameters, Amplitude: 40 % and 80 %; Time: 3, 6, 9 min; Time duration: 7 days; Temperature: 4 °C	HPU treatment ↓TPC by 32.95 % immediately after treatment and 89.20 % after 7 days of storage. HPU treatment ↓TFL by 21.65 % immediately after treatment and 82.81 % after 7 days of storage. HPU treatment ↓DPPH 23.75 % immediately after treatment and 79.50 % after 7 days of storage whereas FRAP ↓ by 27.48 % and 66.03 % after 7 days of storage respectively. In comparison, unprocessed FVJs showed higher retention of bioactive compounds and antioxidant activity, with TFL (46.96 %), DPPH (20.54 %), and FRAP (24.15 %) values significantly higher than those of HPU-treated samples. Overall, HPU treatment substantially influenced the antioxidant capacity of FVJs.	[36]
Grape and Apple	Mixture of juices (50:50)	Frequency: 25 kHz; Amplitude: 70 %; Blanching (thermal process) done at 100 ◦C for 4 min. High-temperature short-time (HTST) done at 72◦C for 15 s. Ultrasonication for 5–10 min. Thermoultrasound (TUS) 5–10 min at 40 ◦C, TUS 5–10 min at 50◦C	Ultrasonication treatment applied for 5 and 10 min ↑ AC viscosity, turbidity, color parameters and anthocyanins concentrations. For bioactive compounds, there were substantial variations in all treatments, no impact on pH or titratable acidity (TA).	[37]
Bayberry	Juice	Sonication power: 600 W; probe tip with a diameter of 13 mm; Frequency: 20 kHz; Amplitudes: 20 %, 40 %, 60 %, 80 %, and 100 %; Ultrasonic intensity levels: 90, 181, 271, 362, and 452 Wcm^2^; Sonication time: 2, 4, 6, 8, and 10 min while the pulse time of 5 s on and other 5 s off. Thermal treatment is done at 90 ◦C for 1 min	Low-intensity US had no effect on pH, TA, yellowness (b*), or Vitamin C. High-intensity US (450 W/cm^2^ for 8 mins) ↑ SOD activity by 21–28 % but ↓ anthocyanins, ↓ bioactive substances and ↓ antioxidant capacity while negatively impacting Vitamin C preservation.	[38]
Strawberry	Juice	Frequency: 20 kHz; 12.5 mm of probe diameter; Sonication time: 5, 10, 15 min. HTST at 72 °C for 15 s. Refrigeration storage at ambient temperature for 14 days.	The US treatment resulted in a 5-log decline of *E. coli* O157:H7 results in ↑ color, ↑ AC and ↑ TPC.	[39]
Black carrot	Juice	Sonication time: 0,4,8 and 12 min; Frequency: 24 kHz	TPC↑, ascorbic acid↑, and AC↑, however, pH, °Brix, viscosity, and turbidity did not change. Where the microbial population was decreased.	[30]
Noni	Juice	Sonication power: 600 W, Sonication time: 25 min, Temperature: 45 °C; Sonication power: 600 W, Time: 10 min; Temperature: 40 °C	US treatment had no effect on pH, acidity, or TSS but ↓TPC and TFL. Additionally, it impacted phenolic compounds, including scopoletin and DAspA/AspA, which contribute to antioxidant capacity. ↑	[1]
Green kiwifruit	Juice	Sonication power: 400 W; frequency: 20 kHz; Sonication time: 0, 4, 8, 16 min.	Cell wall disruption and tearing in tissues ↑, a* and b*, water-soluble pectin, hydroxyl functional groups and AC↑.	[31]
Barberry	Juice	Sonication power 200 W; Amplitudes: 70 % and 100 %, Frequency: 20 kHz; Time duration: 10 and 15 min; Temperature: 25◦C	Sonication treatment resulted in ↓ microbial load, ↑ Total Phenolic Content (TPC) and ↑ Antioxidant Capacity (AC). However, when sonication was applied at 70 % power, it had a relatively minor effect on total anthocyanins, juice color, total phenolics and AC.	[33]
Pumpkin Juice	Juice	Frequency: 25 kHz; Time duration: 10 min; Temperature: 4 ◦C, Sonication power: 400 W	US treatment stabilized juice quality, with ↓ impact on cloud value, carotenoids, color, and particle size and ↑ the juice's rheological properties when compared to untreated control samples.	[40]

Further, among four principal types of cavitation, particle cavitation and optic cavitation are less considered and discussed mechanism for FVJs, majority research focus on acoustic and hydrodynamic cavitation. Although laboratory-scale tests have empirically determined most of the engineering parameters for operating ultrasonication in FVJ industry, field or pilot tests in real environments are still uncommon. Nevertheless, the widespread adoption of this technology in commercial applications has been hindered by its drawbacks, such as metal contamination, inadequate transmission of ultrasonic energy in large volume samples, insufficient spatiotemporal dispersion, low energy efficiency, and greater operational costs.

Demonstration of functional mechanisms of sonication coupled with cold plasma, pulsed electric field and electron beam irradiation and their implications in fruit and vegetable juice matrices, retention of antioxidants and Preservation of enzyme stability which will be reported in different paragraphs:

#### 2.2.1 Sonication cold plasma combined

The present synergistic combination of sonication and cold plasma is the new, non-thermal method of enhancing the quality and safety of fruit vegetable juice. Sonication can make cell walls and membranes more permeable by the mechanism of acoustic cavitation leading to the release of intracellular bioactives like polyphenols and flavonoids. In combination with cold plasma-producing reactive oxygen and nitrogen species (ROS/RNS), the process results in microbial inactivation and retention of thermolabile bioactive compounds. This blend in juice matrices is able to break the cell walls without substantially increasing the temperature and preserving enzymes activity and nutrient value. Cold plasma also has the potential to deactivate oxidative enzyme active such as polyphenol oxidase (PPO) and peroxidase (POD) that cause fast-browning of the juice and antioxidant breakdown. These two methods, therefore, lead to not only greater retention of antioxidants but they extend shelf life, sensory and functional value of the juice.

#### 2.2.2 Sonication combined with pulsed electric field (PEF)

The addition of ultrasonication to pulse electric field (PEF) treatment also increases the effectiveness of bioactive release and inactivation of enzymes in fruit and vegetable juices. PEF also causes electroporation of cell membranes in plants which enhances the physical action of ultrasonic cavitation. This mixture results in an increased mass transfer and I got better extraction of soluble nutrients and phenolic compounds. This synergism helps to break cellular integrity in juice macromatrices leading to higher production of antioxidants such as anthocyanins, carotenoids and vitamin C. At the same time, since this effect produces low temperatures, it minimizes the denaturation of enzymes of beneficial activity (such as catalase and superoxide dismutase), and inactivates enzymes harmful to spoilage, such as PPO and POD. The outcome is a juice product of better functional quality, better enzyme stability, better microbial security and offering the maximum quality that is natural in flavour as well as colour.

#### 2.2.3 Sonication combined with electron beam irradiation

The combination of sonication and electron beam (e-beam) irradiation represents a promising way to improve the retention of antioxidants and at the same time guarantee decontamination of microbes during juice processing. The juice matrix benefits when ultrasonicated upfront in weakening plant tissue physical structures that enhance production of microbubble-mediated shear forces that can penetrate ionizing radiations more conveniently. An addition sterilization process called electron beam irradiation, a non-thermal, high-energy sterilization process further sterilizes the juice by disrupting DNA in microbes and stopping the activity of enzymes. The diffusion of the juice matrix is enhanced when sonication is followed with e-beam irradiation hence making e-beam irradiation to be more efficient. Despite the fact that irradiation in isolation may result in some minor oxidative losses on sensitive antioxidants, both sonication and irradiation can be used in combination to reduce such oxidative losses since sonication contributes positively to antioxidant extractability and also, acting as a buffering agent against oxidation. This two-fold process causes improved retention of phenolic content, improved suppression of browning by enzymes, and longer shelf stability not weened by the thermal degradation that frequently occurs in pasteurization.

This synergy of strategies demonstrates a paradigm change in juice processing-a transition in thinking that previously focused on preservation-based strategies to a holistic approach that aims to improve nutritional and bioactive quality and functional performance of fruit/vegetable juices. Every combination uses distinctive mechanistic advantages to break through traditional-processing constraints with respect to satisfying clean-label and functional-food requirements.

## 3 Irradiation

Irradiation (cold pasteurization) is a non-chemical, non-thermal, and energy-efficient physical disinfectant technique for FVJs that has the potential to enhance FVJs shelf life with numerous benefits over thermal processing[41]. Irradiation includes the revealing of already packaged and massive quantities of FVJs to different resources of ionization energy, such as beams of electrons and electromagnetic waves such as ultraviolet, gamma rays (Cobalt-60) and X-rays[42]. Irradiation with a short wavelength and high intensity is more effective. However, X-rays improve food's shelf life and enhance its quality and safety characteristics and depending on the radiation dose, foods can undergo pasteurization[43]. When compared to heat treatment, irradiation causes little degradation of nutrients and sensory properties in food because there is no rise in temperature during food operations[44]. Irradiating food to high levels of radiation (approximately 10 kGy) enhances microbial quality, rather than increasing toxic effects and without reducing its nutritional properties[45]. Thus, irradiation is a more authentic non-thermal FVJs processing technique due to its ease of availability, low manufacturing cost, and high available energy to convert atoms into ions without causing any radioactivity in FVJs or their packaging. The mechanism of the electron beam irradiation is depicted in Fig. 10.

### 3.1 Types of irradiation

**Fig. 2:**
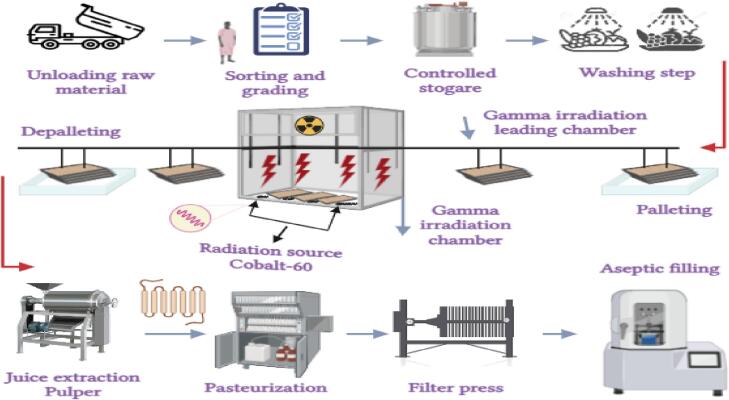
Flow diagram of the fruit and vegetable juice processing steps using a gamma-radiation-assisted extraction system. Adapted from[46,47,48,49]with permission.

The degree of sterilization may differ based on the material utilized, the dose of irradiation, or the type of radiation source. During the irradiation procedure, the FVJs are treated to either ionizing or nonionizing radiation with the goal of extending their shelf life without negatively impacting their nutritional composition[43]. Ionizing radiation is produced when energy is transferred in the form of atomic and subatomic or electromagnetic waves such as gamma and X-rays, or with a greater-energy beam of electrons. In contrast, nonionizing radiation is produced by electromagnetic waves such as UV-rays, microwaves, transparent light, and infrared rays[46]. The flow diagram of the FVJ processing steps using a gamma-radiation-assisted extraction assemblies is depicted in Fig. 2. The subsequent discourse investigates the impact of ionizing and non-ionizing radiation on the quality parameters of FVJs, with a specific emphasis on AC.

#### 3.1.1 Gamma irradiation (γ-ray)

When radioactive isotopes disintegrate, gamma rays are produced, which are high-energy photons. Where, photons of γ-ray offer higher intermittence as compared to UV and X-ray photons. The radionuclides cobalt-60 and cesium-137, which produce gamma rays, are utilized to generate high-energy photons[19]. Both isotopes (cobalt-60 and cesium-137) are routinely employed in food preparation and are able to penetrate into the food to a certain depth[46].

**Fig. 3:**
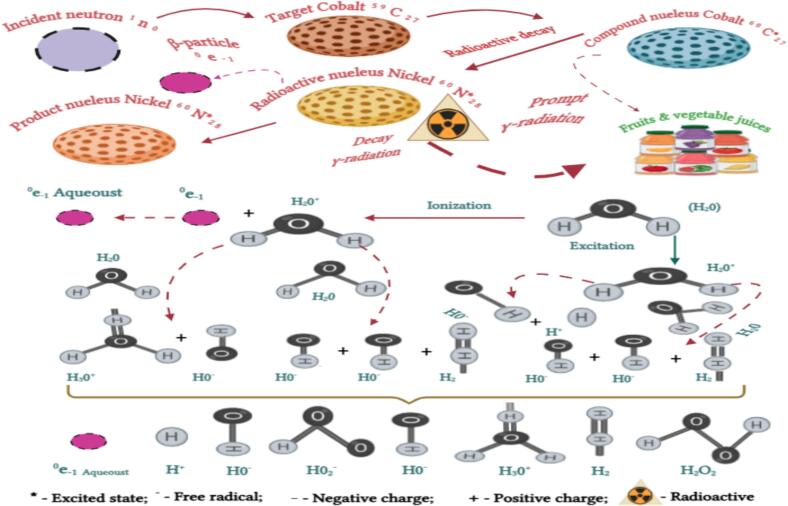
Schematic diagram of gamma-irradiation theory of water molecules radiolysis in fruit and vegetable juice. Adapted from[46,47,48,49]with permission.

However, cobalt-60 is recommended for irradiation over cesium-137 because of its considerable energy, water insolubility, rapid penetration (suitable for bulk food processing), and low starting cost[41]. The schematic diagram of gamma-irradiation theory of water molecules radiolysis in FVJ is depicted in Fig. 3. Sour cheery treatment with γ-ray irradiation has been approved to be effective for enhancement of the overall quality of sour cheery juice, in particular the AC, when the γ-ray dose is not exceeded by 3 kGy[50,51]. A significant improvement in AC has been reported in strawberry and papaya nectar when treated at 2.5–10 kGy[46,52].

#### 3.1.2 X-ray irradiation

X-rays produce ionizing radiation when high-speed beams of electrons clash with metallic elements like tungsten (Ta) and tantalum (W). X-rays have a greater penetration ability than electron beams, which is a critical parameter for selecting a radiation source type. Furthermore, unlike γ-rays, ionizing radiation X-rays could not be produced by harmful isotopes of radiation (cobalt-60, cesium-137)[45]. Moreover, the rising cost of radioactive materials, combined with the consumer's negative impression of gamma-ray-irradiated food, especially FVJs, among consumers, has stimulated demand for new ionizing radiation technologies[53].

However, in the food processing industry, X-rays of energy up to 5 MeV are applied[19]. It has been observed that when apple, orange, and tomato juices were exposed to X-ray radiation, the AC significantly increased while maintaining the original flavor and overall quality[45]. Since quality factors including pH, flavor, acidity, color, and other sensory attributes might impact fruits and vegetables overall quality and customer approval, they should be taken into consideration.

#### 3.1.3 E-beam irradiation

Electron beam irradiation (EBI) is a unique method in which a small dose of ionization radiation is employed to protect the food from the microbial load and the deterioration caused by microorganisms. It also governs the ripening process of fruits as well as vegetables, thereby increasing their shelf life. Fast-speed e-beams have 10 MeV energy and are employed in the food sector for a variety of purposes[19]. Previously, scientists had examined whether these irradiation methods might be used in the production of FVJs[54,23,55]. E-beam irradiation is a novel decontamination and its effectiveness further can be increased by incorporating with other conventional processing, moreover E-beam irradiation is an eco-friendly processing that has no detrimental impact on the product or the environment.

### 3.2 Mechanism of irradiation parameters on AC and other quality attributes in FVJs

The traditional FVJ processing methods are mostly based on the heating process which negatively impact the nutritional profile and other required attributes. However, the effects of irradiation can be achieved without raising the product's temperature. There is no risk of heat-sensitive food components being damaged because the temperature of the food is not increased. The intensity of the irradiation process depends upon various factors, such as the type of irradiation and its intensity, irradiation time, wavelength, kind, and quantity of sample[56]. An electron beam with 10-MeV energy can penetrate to a depth of 39 mm in FVJs with a large amount of water. Whereas γ-rays and X-rays can penetrate into FVJs to a greater depth. As a result, high-energy photons drive the atoms in the target to release high-energy electrons, causing water molecules to split into radicals. Following, UV-C causes biotic stress and produces ROS. As a result, non-enzymatic antioxidants such as glutathione, phenolic, and carotenoids are activated, thus increasing AC[41,51,43].

The FVJs are renowned for their characteristic poly-phenolic secondary metabolites, such as phenolic and flavonoids, which are potent and play a critical role in AC. The role of irradiation on FVJs is contradictory; in some cases, irradiation negatively affects the AC and other quality attributes of FVJs. In this case, a study has shown the impact of **γ**-irradiation on the quality of FVJs phytochemicals and ultimately AC when irradiated at dose levels of 0, 0.4, 1, and 2 kGy. The findings depicted that **γ**-irradiation lowers the phenol content, titratable acidity, anthocyanin, and AC of the FVJs as compared to untreated FVJs[57]). On the other side, irradiation at various wavelengths and doses has shown a significant improvement in phytochemical extraction in different FVJs and, finally, in AC[45,58,59]. Similarly, electron beam irradiation at low doses has no significant effect on AC after processing; however, AC is decreased after storage when treated at low doses[60]. However, a bit higher dose of electron beam irradiation (0.3 and 0.6 kGy) at 1 °C decreased the firmness of FVJs while preserving phenolic content and antioxidant activity. But the phenolic and AC were found to be dramatically reduced when irradiated with a dosage of 0.9 kGy or higher[60]. Another study found that optimum irradiation dosages (0.4 kGy) could increase the preservation time and maintain the quality attributes and AC (of *Citrus unshiu* S. Marcov mandarins). Therein, irradiation at the proper dose could reduce storage losses, enhance the shelf life of FVJs by improving microbial stability, and preserve quality. Further, deoxyribonucleic acid (DNA) can be cleaved directly. These radiations cause the unwinding of DNA and destruction of nucleic acids [19]. Additionally, Table 2 lists the key findings of various irradiation process variables on the FVJs quality metrics. Irradiation has not been approved for use in the FVJs industry due to a lack of authority approval and consumer concerns, although, it is worth noting that consumers have preferences to choose irradiated food over chemically treated food.

**Table 2:** The key findings of various irradiation variables on fruit and vegetable juice quality.

**Source(s) of Fruit**	**Product**	**Conditions for Processing**	**Result(s)**	**Reference**
Pineapple	Juice	UV-C wavelength: 253 nm; Intensity: 1.14 mW/cm^2^; Dose: 3 kJ/m^2^; Time: 4.38 min	UV-C treatment results in carotenoids ↑, protein denaturation↑, and AC ↑ while ascorbic acid ↓	[59]
Orange carrot and celery	Juice	UV-C time:15–60 min	A UV-C treatment results ↑ in AC, ↑ TPC, ↑ TFL and ↑ carotenoid content was observed	[58]
Carrot	Juice	UV-C Flow rate range: 0–7.9 mL/s; Tme: 5–30 min; Dose: 13.2–79.2 J/cm2	No significant changes in total soluble solids, pH, titratable acidity, phenolics, and AC were found	[61]
Mango	Juice	UV-C	Significant increases in phenolic compounds and AC were found, along with improved sensory properties	[62]
Apple	Juice	UV-C wavelength: 275 nm; Dose 200–1200 mJ/cm2	TA, pH, °brix, and reducing sugars remained unchanged. Where TPC ↓ and AC ↓	[63]
Orange	Juice	UV-C wavelength: 254 nm; Time: 15–60 min	No significant effect on pH, TA, TSS (%), ascorbic acid, and AC	[54]
Plum	Juice	Radiation dose: Gamma (cobalt-60) 0.5 kGy	↑ TPC and AC, while no significant effect on color	[55]

Food irradiation has been authorized for usage in over 60 countries across the globe for a wide range of items. Nevertheless, the main obstacle preventing the widespread use of the food irradiation technique is the degree of customer acceptance. From our standpoint, while addressing consumer safety concerns, we believe that using radiation for food preservation will have a similarly positive impact on food safety as sterilizing medical products has had on preventing the transmission of infectious diseases. Therefore, scientists are obligated to assist consumers in comprehending the radiation process and its capacity to enhance our lifestyles and safeguard our well-being.

One form of ionizing non-thermal technology involves electron beam (e-beam) radiation that requires treatment of food products, including fruit and vegetable juices (FVJs), using high energy electrons (usually between 3 and 10 MeVs), generated by electron accelerators. In the case of FVJs, e-beam irradiation has undergone investigation as an approach that has a potential to inactivate microbes and extend the shelf life of product without considerably affecting the nutritional and sensory characteristics of the juice.

It acts by the collision of the accelerated electrons with the microbial DNA and cellular parts that cause the disruption of the replication and metabolic courses which, in turn, promote microbial safety (Sharma et al., 2022). In comparison to thermal approaches, e-beam does not cause high temperatures, and thus can be utilized to maintain heat-sensitive vitamin c, carotenoids, and phenolics, all of which are primary drivers of antioxidant capacity in FVJs (Ramaswamy et al., 2021).

Besides microbial decontamination, it has also been reported that e-beam radiation can also trigger the liberation of bound phenolic compounds due to destruction of cellular matrices thereby possibly increasing antioxidant activity in some instances (Tariq et al., 2020). There are however variations with regard to the type of juice, dose of irradiation, x-ray conditions of exposure. As an example, moderate levels of dose (13 kGy to 3 kGy) usually prove value in decontaminating juice without interfering with its sensory and nutritional properties.

## 4 Pulsed electric fields

**Fig. 4:**
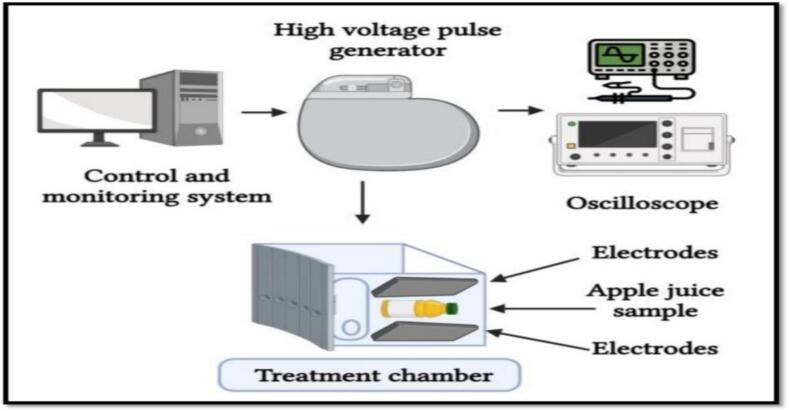
Schematic diagram of pulse electric field[68,47,48,49]with permission.

Pulsed Electric Field (PEF) is a new non-thermal technology for preparing fruit juices. The PEF was first used to preserve food by inactivating microorganisms in the early 1950 s. But it has advanced significantly in recent years and is now broadly employed in the food manufacturing and processing industries. PEF is a non-thermal treatment; FVJs are not heated when they are subjected to PEF; thus, unfavorable changes in FVJs caused by high temperatures are prevented. It's a good substitute for heat treatments for inactivating enzymes and harmful microbes while maintaining the sensory and nutritional properties of FVJs [64]. The growing demand for safe and nutritious food has driven the employment of PEF in food processing[19]. The transfer from small lab and pilot-scale equipment to industrial-scale equipment has happened in recent years. The very first FVJs and smoothies processed through pulse electric fields are now available in several places around the globe, including Austria, Germany, Netherlands, and UK. The PEF's first commercial application began in 2009[65,66]. The PEF is a feasible method and aids in the manufacturing of safe, healthy, and high-quality FVJs[23]. In PEF, a field intensity with a greater pulse rate is administered to FVJs for a short period of time[67]. The field intensity for FVJs is usually between 25 and 85 kV/cm, with an exposure of a few seconds. The block diagram of experimental setup of PEF treatment is depicted in Fig. 4. The mechanism of the pulse electric field is depicted in Fig. 11.

### 4.1 Types of pulsed electric field

The PEF processing further included high-intensity PEF (HIPEF) and mild to moderate-intensity PEF (MIPEF)[69,70]. For HIPEF, electric fields of 10 to 20 kV/cm, or in some cases, 50 kV/cm, were used with a typical pulse width of 2 microseconds to produce an electroporation effect[12,64,70]. In the case of MIPEF, electric fields are mostly applied in the range of 0.9–2.7 kV/cm with a pulse width of 15–20 microseconds for liquid (FVJs) or semi-solid foods that flow freely[71,72]. In terms of AC retention in FVJs, findings were revealed by comparing bipolar HIPEF and single or mono-polar HIFEP treatments, and it has been reported that for better retention of AC in FVJs, bipolar HIPEF is more effective than single or mono-polar HIPEF treatment[12,73,71]. Retention of AC increases in FVJs as frequency (50 to 200 Hz) and pulse width (1 to 7 microseconds) increase but decrease at low electric field strength[19]. However, mono-polar HIPEF treatment has shown a better retention effect of vitamin C in FVJs than bipolar HIPEF treatment, and this might be attributed to the better inactivation of enzymes responsible for the oxidation of vitamin C in mono-polar HIPEF treatment than bipolar HIPEF treatment [12].In comparison, HIPEF and MIPEF treatments are used for electroporation in liquid or semi-solid foods, and bipolar HIPEF was more effective for better AC retention in FVJs, where retention increasing with frequency and pulse width. While mono-polar HIPEF showed better vitamin C retention.

### 4.2 Mechanism of PEF process parameters on AC and other quality parameters in FVJs

The FVJs matrix is a key aspect in determining the PEF process's efficiency. Although pH is an important indicator in the FV juice matrix and can affect PEF performance, moderate PEF has shown the same effectiveness for low- and high-acid FVJs. Suggesting that neither the conductivity effect nor the pH effect PEF performance when moderate-intensity PEF is used. Therefore, it is crucial to accomplish efficient PEF; process conditions can be appropriately adjusted while considering the food matrix to be utilized and the intended goals of the treatments[74].

The most reported work of PEF is related to the inactivation of microorganisms and enzymes. A few studies have demonstrated the benefits and effectiveness of PEF-based phytochemical extraction of its bioactive components with AC properties in FVJs. It is reported that PEF can cause irreversible electroporation of the cytoplasmic membrane of fruits and vegetables, which contains the majority of phytochemicals responsible for AC and other health benefits[65,64,69]. Electric field and pulse strength are critical process indicators for PEF effectiveness. For example, a moderate-field intensity (4 kV/cm) with only a few pulses can result in a higher concentration of phenolic and AC, as well as color, without affecting sensory attributes[65]. However, for stable FVJs storage, they need intensive electric field and pulse strength during PEF (˃ 10 kV/cm), which might negatively affect phenolic and other phytochemicals and ultimately affect AC in FVJs [70].Key findings of various PEF variables on FVJs quality parameters have been depicted in Table 3. Additionally, in HIPEF, more intensive PEF might also result in the production of a higher quantity of reactive oxygen species and/or biomolecule denaturalization to achieve the required outcomes as compared to MIPEF[75]. Therefore, optimized conditions are needed, and further research is recommended to solve this problem.

**Table 3:** Key findings of various PEF variables on the FVJs quality.

**Source(s) of Fruit**	**Product**	**Conditions for Processing**	**Result(s)**	**Reference**
Apple	Unclarified juice	50 pulses; Voltage: 30 kV cm^−1^; No. of cycles: 4, 6, 8 (total pulses: 200, 300, and 400); Temperature: < 35 ◦C for 24, 48 and 72 h	No effect on ascorbic acid and TPC, but food spoilage microflora↓ and AC↓.	[68]
Pomegranate	Clarified juice	Extent of pulse: 3 µs pulse Postpone time: 20 µs frequency: 500 pps controlled flow rate: 60 mL min − 1. Experiment design (DOE): 0, 17, 23, 30 kV cm − 1 5, 15, 25, 35 ◦C Total duration of treatment: 108.4 µs, with related energies of 37.5, 50.3, and 65.3 J.	Titratable acidity ↑, color (a*, b*, and L*) ↑, TPC↓, AC↓, anthocyanins↓, sourness↓, sensory properties↓, and ascorbic acid ↓	[76]
Red grapes	Juice	5 kV/cm, 40 microseconds 4 ◦C, 10 ◦C	TPC↑, anthocyanins↓, AC↓, and color index↓.	[75]
Kiwi and carrot	Juice	22.22–56.56 kV/cm, 2400–4800 microseconds	No changes in ascorbic acid, where TPC ↓ and AC↓	[74]
Orange	Juice	35 kV/cm for 1500 microseconds	Improved AC and color properties	[69]
Broccoli	Juice	35 kV/cm, unipolar pulse, 500 microseconds, 25 kV/cm, bipolar pulse, 1250 microseconds	Retention of ascorbic acid is between 90.1 % and 67 %, preserving bioactive compounds and AC.	[77]
Watermelon	Juice	35 kV/cm, 50–250 Hz, 1–7 microseconds	Ascorbic acid↑, lycopene↑, and AC↑	[12]

The major components of PEF equipment involved in FVJs processing are a treatment chamber, a high-voltage power supply, a control system, a pulse generation unit, and a cooling system. During treatment, when the temperature of the equipment increases (but its highest temperature is 40 °C, which is much lower than heat-sensitive conditions) due to joule heating, the cooling system balances it by providing a cooling effect[12,74].High voltage in PEF with moderate to higher intensity field strength can break the cell membrane, cause cells to swell and break, and increase AC[78]. The FVJs are placed in the chamber for treatment between two electrodes of stainless steel, and greater power pulses of 50 kV/cm are applied for a limited time (microseconds to milliseconds)[79].

The PEF works on the principle of a hybrid of electroporation and electro-permeabilization and induces changes in the structural integrity of tissues. For instance, when a fruit, vegetable, or bacterial cellular membrane is exposed to an electric field, it causes disturbances in the cell wall and results in the creation of holes in the cytoplasmic membrane (fruits and vegetables) and pores in the cell membrane (bacteria)[64]. It is called electroporation. By electroporation, PEF improves electro-permeability (cellular structure destabilizes as a result of electroporation, leading to a rise in cell permeability) and increases intracellular release of bound-form phytochemicals[65]. Further, these compounds can come out of the cells easily due to mass transfer, thus increasing the concentration of these phytochemical compounds in the solution and resulting in better AC[75,72].Further, PEF's effectiveness is determined by significant processing parameters such as field strength, pulse width, pulse frequency, treatment time, energy, polarity, electric conductivity, and applied temperature. While Bhattacharjee et al. [12] have found PEF processing for FVJs to be less successful than simple microbial suspensions, this is due to the fact that FVJs have more macronutrients (lipids and proteins). Further, the medium's electric conductivity is also essential, as FVJs with high conductivity produce small electric currents that are unsuitable for PEF processing. However, it is also reported that the efficiency of PEF in FVJs can be improved by the existence of natural antimicrobial compounds.

In addition, some primary elements that influence the PEF effectiveness of FVJs are as follows: (a) pH, (b) water activity (aw), and (c) TSS[12]. Overall, PEF has been successfully applied for FVJ processing where it does not show in the alteration of major functional compounds. Nevertheless, in a specific context for the FVJs industry, there are several obstacles/limitations to PEF's successful and efficient commercial implementation, such as some electrical parameters (pulse frequency) and the presence of halides in the food matrix, which can accelerate the release of metals (Ni, Fe, Mn, and others) from the electrode due to their corrosive properties and can easily mix into the FVJs[73]. However, this attribute might be controlled or minimized by using carbon electrodes, but further research is still recommended to optimize its condition and lower the risk of these electrodes’ constituents mixing into the FVJs.

Moreover, PEF-related mechanisms, kinetics, and chemically stable optimal process parameters conditions for enhanced quality, safety effect (microbial and enzymatic inactivation), intracellular extraction and their complex interaction with food components (to achieve better extraction of phytochemicals), and improving the retention of AC along with other functional properties are some factors that need to be considered.

In addition, during standard PEF processing, electrochemical reactions inevitably occur at the electrode-food interface within the PEF treatment chamber. In order to ensure the successful commercialization of PEF, it is crucial to minimize reactions that lead to corrosion and fouling of the electrodes, electrolysis of water, migration of electrode material components, and chemical changes of food products. These reactions have the potential to negatively affect safety, quality, process efficiency, equipment reliability, and cost considerations.

## 5 Atmospheric cold plasma (ACP)

The atmospheric cold plasma, or ACP, is a highly significant and innovative non-thermal food preservation method. Compared to other non-thermal technologies, ACP has numerous benefits and can be applied to FVJs processing[80,81]. To create plasma (ACP), a gas (CO_2_, O_2_, N, He, Ar, or air) is ignited at room temperature in a high electric field. Chemically reactive entities (charged particles, reactive oxygen and nitrogen species/radicals (RONS), heat, and UV radiation) are agitated during the processing, and these species vary depending on the characteristics of plasma process parameters such as plasma generation method, frequency, flow rate, voltage, bubble size, reactor shape, and the kind of gas employed [82,83]. The appropriateness of this novel ACP processing is supported by its high efficiency to produce safe, stable, and nutritious food and the avoidance of undesirable changes in FVJs associated with thermal pasteurization. And it has the potential to replace or at least supplement traditional pasteurization procedures. It is economical, non-toxic, eco-friendly, leaves no waste, and has a minimum impact on FVJs quality loss[83].

### 5.1 Types of plasma source

**Fig. 5:**
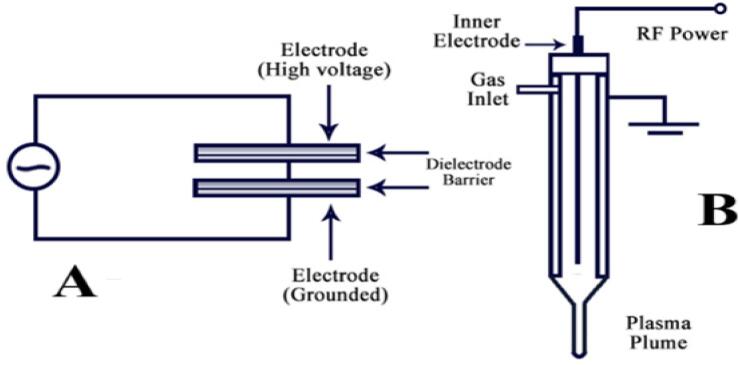
Schematic drawing of direct barrier discharge and (A) atmospheric pressure plasma (B) jet plasmas[84]with permission.

The plasma has been categorized based on its excitation mode (which can be generated in a variety of ways), including coronas, dielectric barrier discharge (DBD), glow discharge plasma, microwave discharges, and plasma jets Fig. 5 **(A-B)** [80,84]. In this review, we will not discuss many details about its kinds, as we have mentioned many details in our previous study as well as in some other studies presenting good insights on different types used for plasma generation.[85,47,48,49]. Researchers had given preferences for specific devices of plasma on the basis of their applications in food, in particular FVJs. It's worth noting that different plasma devices produce different reactive gases or species, and these species all vary depending on their device. Each plasma device performs its own specific function and has different applications.

One plasma device may not be successful in the application performed by another plasma device. A high-voltage electric cold plasma (HVCP) device based on a DBD plasma source has played a vital role in a wide range of FVJs processing[86,87,47,48,49,88].

#### 5.1.1 Dielectric barrier discharge (DBD) plasma generation

**Fig. 6:**
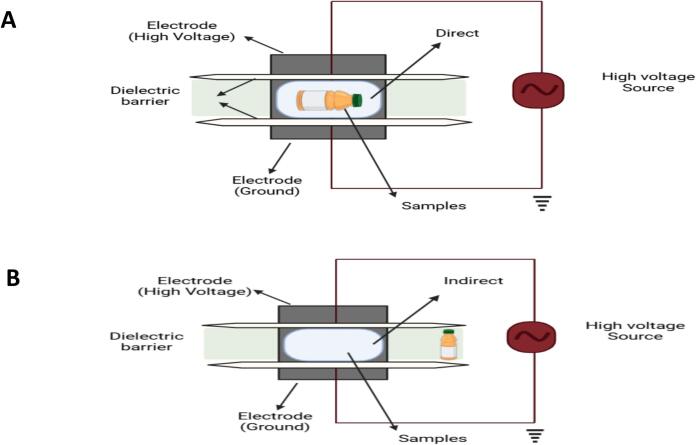
Schematic diagram of (**A**) direct and (**B**) indirect DBD plasma treatments on liquid samples adopted from[47,48,49]with permission.

It is a type of direct current discharge used for FVJs processing. At their discharge area, one dielectric barrier must be inserted that is either directly or indirectly treating the FVJs sample (Fig. 6 **A-B**). The DBD is one of the most extensively utilized plasma because it can operate at a variety of frequencies and power supplies. It uses different kinds of sources for gas, resulting in the most uniform plasma dispersion with a significant range of short-lived discharges. Furthermore, DBD is also known as self-pulsed discharge because the discharge current cannot exceed a level that produces arcing[86,47,48,49]. In order to produce atmospheric pressure non-thermal plasma, an extremely strong field of electric strength is necessary to induce a collapse across the discharge gap of the dielectric barrier discharge device, which ranges from millimeters to centimeters. In this scenario, the size of the FVJs sample is fixed[86,89], Ozen, Adhikari et al. 2024).

Cold plasma (CP), also known as non-thermal plasma, is a new technology used in food processing which consists in the formation of reactive species at non-thermal temperatures owing to the ionization of gases like air, oxygen, nitrogen or helium. It generates a complex combination of reactive oxygen species (ROS), reactive nitrogen species(RNS), UV photons, electrons and charged particles, which combine with the surface area of food materials such as fruit and vegetable juices (FVJs) (Misra et al., 2016).

##### 5.1.1.1 Impact on phenolic compounds

Cold plasma may influence phenolic content in FVJs in two conflicting processes: Degradation of phenolic compounds: In FVJ, high-energy ROS (e.g.: hydroxyl radical, singlet oxygen) produced during exposure to plasma can oxidize and degrade part of the phenolic compounds, especially phenolic compounds susceptible to oxidation (e.g. anthocyanin, flavonoid). So this could result in the loss of total phenolic content (TPC) when not optimized in modes of treatment (time, voltage, gas composition) (Sarangapani et al., 2017). Increased releasing and extraction: On the other hand, CP has the ability to disrupt cell walls and vacuolar membranes leading to more release of the bound phenolic compounds present in cell matrices into the juice matrix. The solvent becomes more available since lignocellulosic and pectin-containing material is broken down structurally, which results in increased amounts of free phenolics that can be measured (Ma et al., 2015). In this way, the net result of phenolic content is dose- and matrix-dependent since modest plasma treatments can increase phenolic retrieval, but high doses can ultimately result in degradation.

##### 5.1.1.2 Effect on antioxidant capacity (AC)

CP can affect the AC of antioxidants mainly through phenolics, carotenoids, and vitamins, which condense in the following forms: Encapsulation of antioxidant enzymes: By using cold plasma, some indigenous antioxidant enzymes, such as peroxidase, superoxide dismutase, and catalase, can be activated, especially in the case of whole tissues or freshly extracted juices, temporarily enhances AC (Ji et al., 2020). CP is an effective non-thermal technique that can extend the shelf life of pineapple juice stored in glass bottles for up to 90 days under refrigerated conditions [90].

##### 5.1.1.3 Induction of abiotic stress

The oxidative stress caused by CP treatment triggers plant defense mechanisms and phenolic metabolite production through activation of the tissue level. Such accumulation of phenolics due to abiotic stress has been reported specifically in a situation where raw plant files have been treated before juice extraction[13]. Stability of antioxidants: Practiced in CP, thermal processing considerably increases the temperature, destroying labile antioxidant nutrients like vitamin C and carotenoids and can negatively affect the total AC (Ziuzina et al., 2020). Formation of oxidized products: In certain circumstances, excessive usage of CP could contribute to oxidation of phenolics and vitamins and the resulting AC might be reduced when reactive species surpass the antioxidant levels.

#### 5.1.2 Radio frequency plasma generation (RF)

**Fig. 7:**
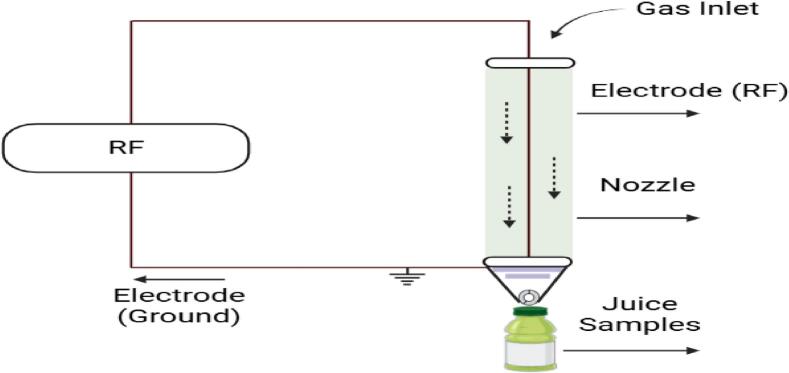
Schematic diagram of RF plasma adopted from [95]with permission.

It is produced with the help of a generator. The generator provides the energy that helps in the ionization of gas. High voltage and high frequency are applied to it. The RF-plasma that is generated has a jet-like appearance and can be sprayed directly to the FVJs surface (Fig. 7).Power produced by the generator, distance, and flow rate of gas all influence the intensity of plasma[86,91]. RF-plasma is easier to utilize than conventional plasma treatments because it takes less treatment time and does not require a vacuum chamber[92]. Further, DBD’s have a space limitation; hence, RF plasma can create plasma in free space, which is a better and more suitable choice for processing. Commonly, noble gases, nitrogen, and air are used as carrier gases to ignite plasma. While air plasma jets are most widely used in FVJs[93,94]. However, RF-plasma application in FVJs processing is relatively limited, and to accomplish favorable treatment, RF-plasma assay co-working and constant movement of FVJs are necessary.

#### 5.1.3 Microwave plasma generation

Microwave generators induce plasma without the use of electrodes, unlike plasma generators, as discussed above. A waveguide transports power in the microwave area, and an impedance matching tuning component is included. The operational gas is injected into a nozzle, which is situated where the electric field is strongest. The microwaves have a frequency of GHz, resulting in exceptionally high-temperature microwave plasma due to the huge energy input[96]. The plasma-processed gas must pass through a cooling system before being collected at ambient temperature in a concentration chamber, ensuring non-thermal reactivity in the reaction chamber with FVJs[97,98]. The inclusion of a cooling system, on the other hand, will raise the processing cost. In addition, low-pressure microwave plasma, microwave plasma UV lamps, and electron cyclotron resonance microwave plasma are major classes of microwave plasma. Some important advantages of microwave plasma are better electron density, higher efficiency in producing reactive species, and the fact that it also provides significant results in terms of time-saving, low cost, non-toxic nature, and contamination-free non-thermal FVJs processing.

**Fig. 8:**
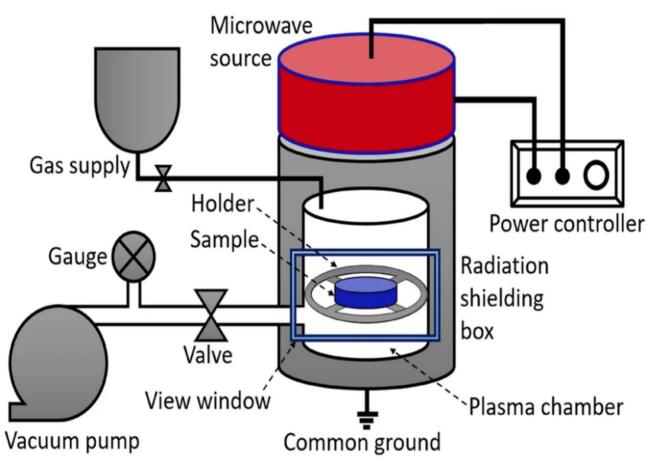
Schematic diagram of a typical microwave plasma treatment system adapted from[99]with permission.

A schematic diagram of a typical microwave plasma treatment system has been enlisted in Fig. 8. Whereas a microwave plasma UV lamp can produce UV light at 254 and 185 nm, however, microwave plasma UV lamp application in the FVJs industry is also limited, and not many studies have shown the impact of microwave plasma UV lamps on the antioxidant capacity of FVJs. Next, electron cyclotron resonance microwave plasma has advantages over other microwave plasma in that it has a low average electron temperature (5–10 eV), higher ionization, and flexible temperature and pressure conditions[96,99]. In the case of electron cyclotron resonance microwave plasma, much work has focused on its germicidal effect, sterilization of food packaging material, and deposition of barrier coatings for packaging material by microwave plasma rather than the antioxidant capacity of FVJs. Therefore, the details of the mechanism of microwave-based plasma for FVJs are still not well exploited and need to be further considered.

### 5.2 Effect of high voltage cold plasma on AC and other quality parameters in FVJs

The impact of any processing treatment or procedure on the AC of FVJs is a critical quality indicator in juice processing[80,7,32,81]. Antioxidants and bioactive compounds have the ability to remove free radicals that are harmful to the human body, as well as minimize the menace of most illnesses caused by oxidative stress[80]. The FVJs contain tocopherol (vitamin E), vitamin A, phenolic components, and ascorbic acid, which are accountable for AC and DPPH free radical scavenging, and are dietary antioxidants found in plant sources. High-voltage cold plasma, or HVCP, has been apt for FVJs processing and has shown positive hope as it has the capability to release bound forms of phenolic and thus can improve the AC of the FVJs[80,34,81].The HVCP method was applied to evaluate the antioxidant capacity of apple, orange, and white grape juices[100,101]. Key findings of HVCP-treated FVJs and its overall impact on the AC are shown in Table 4. It can be seen that the HVCP treatment at 80 kV for two minutes or more (increasing the treatment time) results in a decline in AC and free radical scavenging in FVJs. This could be caused by the low levels of total phenolic found after the HVCP treatments. However, this reduction of DPPH free radical scavenging ability and the overall AC effect in FVJs were more significant in thermal pasteurization[101]. Hence, the juice industry has the potential to produce stable, nutritious, and healthy FVJs to fulfill customer demand. The mechanism of the cold plasma discharge is depicted in Fig. 12.Table 5..

**Table 4:** Key findings of various HVCP variables on FVJs quality.

**Source(s) of Fruit**	**Product**	**Conditions for Processing**	**Result(s)**	**Reference**
Kiwi	Juice	Voltage: 24–36 kV; Frequency: 50 Hz; Time: 0–10 min; Sample: 2–10 mm	No effect on TSS, pH, and TA, but ascorbic acid↓, AC↓, TPC↓, and TFL↓	[9,10]
Orange	Juice	Voltage: 70 kV (kilovolts); Frequency: 50 Hz; Gas: Air; Sample: 20 mL; Control sample: Raw juice; Time duration: 20–40 sec;	HVCP results in stable pH with minimal discoloration. Whereas TFL↑ and AC↓	[101]
Apple	Juice	Power: 30 W, 40 W, 50 W; Frequency: 10 kHz; Gas: Air; Sample: 3 Ml; Control sample: Raw juice; Time duration: 0–40 sec	There is no fluctuation in the Brix, TPC and AC measurement. But acidity↑, a* and b* values↑ and pH and L* value↓	[100]
White grape	Juice	Voltage: 80 Kv; Frequency: 60 Hz; Gas: Air; Treatment in a container. Control sample: Raw juice; Time duration: 1–4 min	The AC ↑ in comparison to the control sample.	[102]
Blueberry	Juice	Voltage: 11 kV; Frequency: 1000 Hz; Gas: argon at a concentration of 0.5 % and 1 % oxygen. Control sample: Pasteurized juice; Gas flow: 1 L/min; Time duration: 2–6 min	Anthocyanins ↓, ascorbic acid↓, AC↓, and overall color changes↓ TPC ↑.	[82]
Cloudy apple	Juice	Voltage: 8–10.9 kV; Frequency: 20–65 kHz; Gas: Air; Sample: 10 mL. Control sample: Raw juice; Time duration: 1–5 min; Refrigration storage: 1–4 weeks	Brightness↑, TPC↑, AC↑, non-enzymatic browning↓,pH↓, with no polyphenol oxidase (PPO) residual activity.	[103]
Tomato	Juice	Arc discharge; Gas: N_2;_ Voltage: 3.8 kV; Power: 40 W; Time duration: 30–300 sec, 440 L/h, 1 cm	Lycopene↑, AC↑, and carotenoids↑, and vitamin C↓.	[104]
Acerola	Juice	Gas: N_2_-glow plasma; Frequency: 80 kHz, Sample: 10–20 mL/min; Time duration: 5–15 min	Vitamin A ↑ and carotenoids↑ vitamin C↓, TPC ↓, and AC↓	[105]
Cashew apple juice	Juice	Gas: Indirect cold plasma; Frequency: 80 kHz, Sample: 10–20 mL/min; Time duration: 5–15 min	A reduced flow rate of N_2_ plasma ↑ vitamin C, ↑ TPC, and ↑ AC. AC ↑ proportionally with the ↑ in N_2_ flow up to a certain point, after which it begins to decline.	[106]
Apple orange, tomatoes and sour cheery	Juice	Gas: Air, 3000L/h, Power: 650 W; Time duration: 30–120 sec	TPC ↑ but pH and color index have remained constant.	[107]

**Fig. 9:**
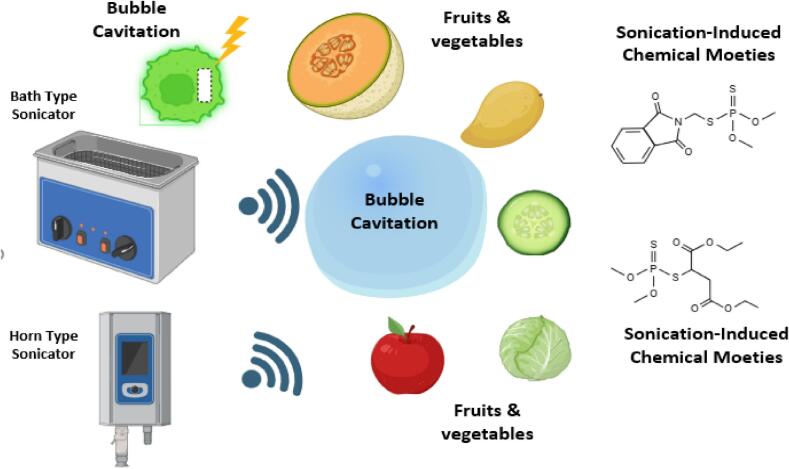
Mechanism of ultrasound assisted extraction on fruits and vegetables juices.

**Fig. 10:**
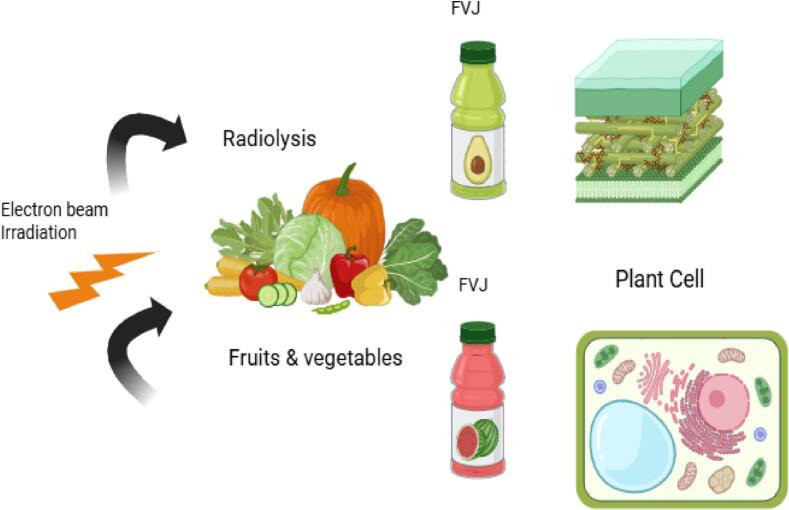
Mechanism of E-beam irradiation on fruits and vegetables juices.

**Fig. 11:**
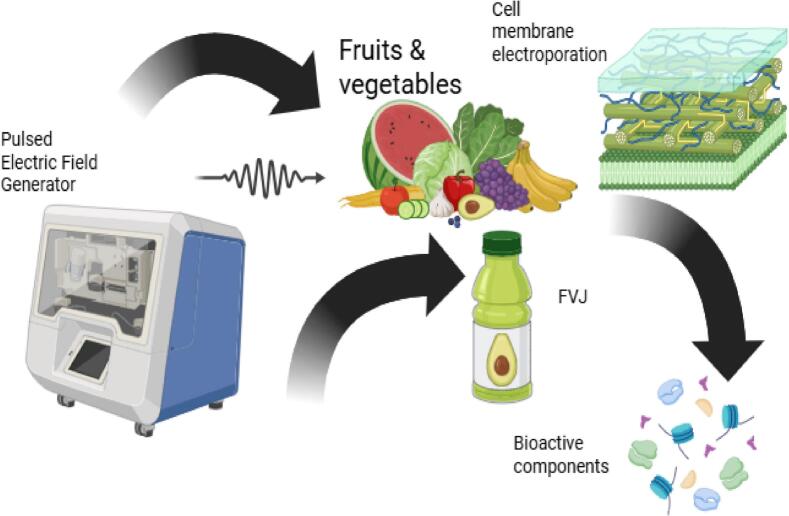
Mechanism of pulsed electric field on fruits and vegetables juices.

**Fig. 12:**
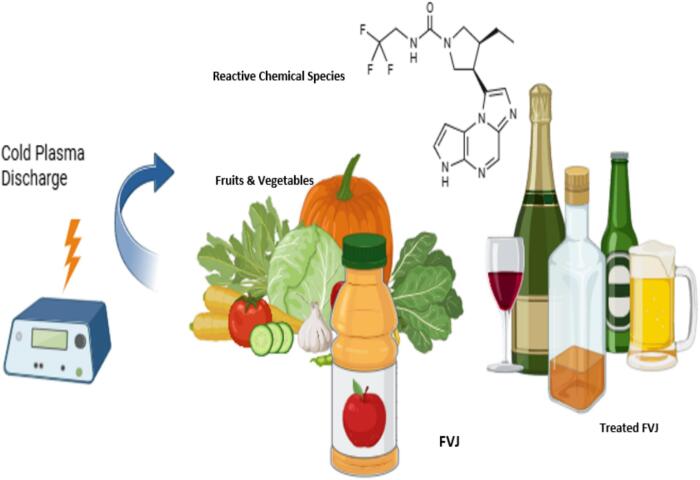
Mechanism of cold plasma discharge on fruits and vegetables juices.

**Table 5:** Comparison of non-thermal techniques for key quality effects of fruits and vegetables (FVJ) juice matrix.

**Method**	**Year**	**Juice Matrix**	**Conditions**	**Key Quality Effects**	**Shelf Life / Sensory Outcome**	**Reference**
**Ultrasound**	2015	Raspberry & Blueberry puree	45 kHz, 100–200 W, varying time	↑ Total phenolics & antioxidant activity; improved viscosity/rheology	Improved quality vs control	[108]
**Ultrasound**	2018	Mixed spinach–fruit juice	US-assisted extraction (long time)	↑ Carotenoids, phenolics; improved color; no major change in pH/TSS	Enhanced color and antioxidant content	(Khandpur,, & Gogate, 2018)
**Ultrasound**	2021	Apple/carrot/blueberry juice	Various 20–40 kHz protocols	↑ Ascorbic acid, phenolics; antioxidant activity; improved color/viscosity; minimal pH/TSS impact	Shelf life extended compared to thermal pasteurization	[109]
**Cold Plasma**	2017	Cashew apple juice	DBD atmospheric plasma (∼30 kV, minutes)	↓ Microbial load; good retention of vitamin C; moderate phenolic reduction	Safer product with maintained sensory quality	[106]
**Cold Plasma**	2019	Strawberry, kiwi, apple juice	Various DBD/jet setups 2015–2019	Microbial inactivation up to ∼ 7–log; slight vitamin C loss (∼15–22 %); enzyme inactivation; color retention	Nutritionally superior and sensory stable	[110]
**Cold Plasma**	2023	Orange juice	DBD pasteurized plasma treatment	Modified volatiles/aroma; mitigated off–flavors; minimal pH/TSS/color change	Superior antioxidant-rich quality	[111]
**Cold Plasma**	2024	Apple & cantaloupe juices	Atmospheric cold plasma	Maintained volatile quality; preserved antioxidants; improved microbial safety	Refrigerated shelf up to 105 d; room temperature up to 45 d	[112,113]
**Cold Plasma**	2024	Kiwifruit juice	DBD optimized vs extreme (2024 study)	Particle–size reduction, increased homogeneity; microstructure disruption; sensory stable	Sensory acceptable; extended shelf life under mild PL	[9,10]
**Cold Plasma**	2021	Various fruit juices	Wide DBD/jet protocols	Summarizes microbial inactivation (0.15–7.4 log), enzyme effects, nutrient retention	Enhanced physical quality; shelf-life benefits	[85]
**Cold Plasma**	2023–24	General fruit juices	Multiple CP systems	Notes higher antioxidant activity post–treatment; detailed kinetics and storage effects	Enhanced juice quality and healthier profile	[9,10]
**PEF**	2015	Grapefruit juice	0–25 kV/cm, 600 µs, 40 °C	↑ Total phenolics, TAC, carotenoids, DPPH; ↓ viscosity; no change in pH/TSS/color; microbial reduction	Improved quality vs control	[114]
**PEF**	2016	Pinot Noir grape juice	∼35 kV/cm, pulses	↑ anthocyanin release; improved polyphenols and health-promoting properties	Enhanced color and antioxidant content	[115]
**PEF**	2017	Date juice	high-intensity PEF vs heat	Better retention of vitamin C and phenolics; microbial deactivation	Shelf life extended compared to thermal pasteurization	[116]
**PEF**	2019	Strawberry purée	validated scale-up PEF pasteurization	Effective microbial inactivation (≥5–log); quality retention	Safer product with maintained sensory quality	[117]
**PEF**	2021	Blood orange juice	mild PEF (≤45 °C maintained)	Full vitamin C retention (∼62.9 mg/100 mL); improved flavanone rutinoside; no thermal damage	Nutritionally superior and sensory stable	[118]
**PEF + US**	2021	Spinach juice	US at 40 kHz/200 W, 21 min + PEF at 9 kV/cm	Max levels of flavonoids, phenolics, carotenoids, chlorophyll, vitamin C; strong enzyme inactivation	Superior antioxidant-rich quality	[119]
**PEF**	2020	Strawberry juice vs thermal	18.5 kV/cm, 500 µs, 100 Hz for 60–90 s	Similar pH, TSS, color to fresh; high vitamin C; effective microbial stability	Refrigerated shelf up to 105 d; room temperature up to 45 d	[120]
**Pulsed Light (PL)**	2023	Sweet lime juice	Pulsed light	Minimal change in pH/TSS; better retention of phenolics; some vitamin C degradation at higher fluence	Sensory acceptable; extended shelf life under mild PL	[121]
**Pulsed Light (PL)**	2021	Tomato & mango slices	PL pulses 2–15 J/cm^2^	Reduced browning, maintained firmness/color; increased antioxidant levels	Enhanced physical quality; shelf-life benefits	[122]
**PEF**	2020	Blueberry juice & by-products	PEF pretreatment before pressing	↑ extraction yield and antioxidant activity; improved phenolic recovery	Enhanced juice quality and healthier profile	[123]

The application of HVCP treatment enhanced the AC of white grape juice and cashew apple juice. Researchers have suggested that the elevated phenolic content of white grape juice and apple juice, caused by the partially inactivated polyphenoloxidase, may lead to an increase in AC[101]. Further the AC, color, and TPC of apple juice saw considerable alterations following plasma processing. The apple juice was greatly influenced by the frequency of plasma excitation. The excitation frequency in dielectric barrier discharge plasma systems can vary between 50 and 30,000 Hz. When apple juice was exposed to stimulation frequencies of 50 Hz and 200 Hz, there was a noticeable rise in both the AC and TPC. However, the color of apple juice was slightly impacted[124]. Nevertheless, it may also lead to a decrease or no observed alterations in the AC in orange juice [27]or probiotic juice made from orange and kiwi fruits when subjected to the HVCP treatment[125].

The alterations in tissue stress response, endogenous antioxidants, and enzymatic activity induced by HVCP all impact antioxidant efficacy. Prior studies have demonstrated that when antioxidant chemicals persist at a high concentration after plasma treatment, their antioxidant properties are also sustained at a high level[126]).The decline in antioxidant levels is believed to be counterbalanced by the tissue's stress response to HVCP treatment[98].Various studies have proven the overall effect of HVCP for AC enhancement through better inactivation of polyphenoloxidase (PPO) and enhanced extraction capacity of total phenolic compounds; however, some studies did not demonstrate a similar phenomenon in the case of probiotic orange and kiwi juices. This conclusion shows that the diversity in the AC impact is caused by the creation of many plasma species that glow discharge plasma induces[127,106]. More research is required to fully comprehend the chemical makeup of plasma species, their interactions with AC and other relevant substances, and the cytotoxic effects of these interactions.

The literature focuses on the growing exploitation of HVCP, which is at present in the testing stage in labs, as a promising non-thermal method for FVJs. It has obtained approval for its substantial upside throughout various domains and shows outstanding potential for commercialization in the FVJs industry. However, the efficacy of HVCP is contingent on regulating several influential aspects to attain the intended results. While HVCP has promise in offering numerous benefits, it is important to also take into account any potential negative consequences.

## 6 Current challenges and future directions

### 6.1 Future trends for NTTs in FVJs and other beverage industries

Future research is needed for scale-up production of non-thermal-treated FVJs and other beverages, and the following aspects should be addressed: optimal process parameters, action mode for AC retention, cost-effective machine design, equipment safety and regulatory approval, and consumer acceptability.

Previously, much work has been reported and confirmed to enhance the effectiveness and efficiency of antioxidant compounds in non-thermal-treated FVJs, but the enhancement mechanism remains unclear. So deep research should be focused on understanding the biosynthesis of metabolites involved in enriched Kyoto Encyclopedia of Genes and Genomes (KEGG) pathways and antioxidant metabolism-related enzymes and their gene expression. Also, the toxicity and chemical residue evaluation of reactive oxygen and nitrogen species produced in non-thermal-treated FVJs is needed. The outcomes of these will assist in process optimization and its successful commercial implementation.

Since FVJs are considered potentially unsafe foods because of having low acidity (pH range between 5 and 6) and are highly perishable as well as susceptible to contamination if not handled properly. Therefore, process optimization and validation are crucial for the successful implementation of NTTs in the FVJs industry. Unfortunately, there are no defined methods to validate and regulate these NTTs to attain the desired outcomes for FVJs processing. For example, in HVCP at the industrial scale, electrical and optical components are the process control parameters. Electron density, voltage, power, and ion species are usually considered to observe the electrical component, while optical density can provide information for reactive species by optical emission wavelength. It's important to note that there is currently little to no research on the electrical or optical components of HVCP in relation to FVJs or other beverages. Whereas, in the case of ultra-sonication, low power with a short duration or low power with an extended duration are found to be more optimal parameters for AC in FVJs. Therefore, the necessity for additional investigation emerges, which is legitimate and warrants resolution through research.

To actualize the production of industrial-scale non-thermal treated FVJs, the nature of the food product, its variety, and other geometry should be considered. The optimized process should meet the maximum acceptability of different natures, varieties and states of foods and should be able to process food continuously without causing any physical injury, regardless of its size, shape, or kind. Keeping in view all the requirements and characteristics necessary to achieve the above-mentioned goals, the design and the machine cost should be rational and acceptable for their commercial application. Furthermore, the safety and worker health aspects cannot be ignored and must be addressed properly to meet regulatory acceptance and approval, as regulatory approval is the major hindrance for NTTs in the FVJs and other beverage industries. Therefore, sufficient measures should be placed to ensure the safety of the workers, such as installing sensors to control strong electric and magnetic fields and gas leakage, limiting the worker's exposure to reactive species and radiation, and using proper safety and recommended clothing for the radiation zone. Furthermore, proper disposal of RONS is essential to prevent their release into the environment. Ultimately, the successful implementation and acceptance of any technology, method, or product is contingent upon its end user's acceptance. There are some reports about the application's societal and end-user acceptability and views, its implementation benefits, and the NTT-associated risks.

However, there is a need to expand further investigation of these NTTs in the FVJs industry, along with prompt and effective communication for their early adaptation stage. Principally, scientists, engineers, industrialists, distributors, experts and consumer groups should work with regulatory bodies in optimization, evaluation, implementation, and developing guiding documentation to speed up the implementation process and to accelerate the commercialization of these NTTs.

### 6.2 Commercialization of NTT-Based systems for industrial use

The capabilities of various NTTs in AC retention or activating the antioxidant system show that so far there is no or little effect of these NTTs on the physicochemical properties and particularly AC of FVJs as compared to commercially available thermal pasteurization, which needs to be acknowledged by food scientists and engineers. Although further research is needed to exploit the advantages of these novel NTTs before these technologies can be launched at a commercial level. In addition, understanding the biosynthesis of antioxidant metabolism involved in Kyoto Encyclopedia of Genes and Genomes (KEGG) pathways and major antioxidant-related enzymes, i.e., superoxide dismutase (SOD) activity, ascorbate peroxidase (APX) activity, catalase (CAT) activity, and antioxidant enzyme-related gene expression levels, i.e., FaCu/ZnSOD, FaMnSOD, FaAPX, and FaCAT, should be studied and discussed in detail.

Lately, regulatory approval has depended on the results of the verification and validation processes. Therefore, the implementation of an effective reporting system is necessary to facilitate easy, independent, and reproducible verification and validation processes. As it will provide a fundamental concept that uniquely aligns non-thermal technologies as an opportunity for system modification for researchers, industrialists and other associated alliances, with aims to achieve efficient, easy scale-up, versatile, require expertise in experimental design, interaction with food components, and efficient energetic capacity technology, along with hands-on experience of the reaction mechanism for coupling prospects for present and future economic and social needs.

### 6.3 Assessing the impact of NTTs on the FVJs Industry: Scientific, Social, and economic Perspectives

The successful implementation of NTTs in the FVJs industry will create new knowledge and pave the way to develop green and sustainable, minimally processed high-quality food products, which will be beneficial for establishing fundamental theories, attracting, developing, and nurturing businesses to develop superior, safer, higher quality products, with decreased negative associations with products.

The implementation of NTTs in the FVJs industry will fit well with sustainable strategies around the globe. Considering the sustainable, green impact on human health, social and technological progress, and sustainable production and consumption, these NTTs are chemical-free methods for FVJs preservation and thus are environment-friendly, technological methods that are not only beneficial for researchers and industries but also for society by fulfilling their needs and improving their health.

The successful implementation of NTTs in the FVJs industry will give companies a competitive advantage, leading to a bigger market share, increased investment in product development and infrastructure, and more employment in R&D. There will also be a significant economic impact from attracting, developing and nurturing businesses, scientists, and talented people. These NTTs have a strong strategic position in driving technological development and food processing.

Conversely, certain characteristics impede the successful implementation of NTTs at the industrial level. Such as a poor understanding of the impact of NTTs on food ingredients and its physiochemical properties (i.e., antioxidants). Involvement of strong electric and magnetic fields, free radicals, and reactive species, lack of process optimization, difficulty treating liquid foods in bulk amounts, scarcity of studies about their societal and economic impact, and complications in regulatory approval are some of the other negative aspects that are hindrances to its regulatory approval, industrial adaptation, and consumer acceptability.

In addition, each NTT (non-thermal technology) has its own advantages and disadvantages. Some face challenges in evenly spreading the desired impact throughout the entire liquid volume of FVJs, especially in large volumes. This limitation hinders their widespread use in industrial settings. On the other hand, certain NTTs exhibit noticeable inconsistencies in terms of quality and safety concerns. Some non-traditional trading platforms encounter difficulties in establishing trust with their customers. So, it's important to look at how the quality features change and what the pros and cons of FVJ are during the NTT processes while still getting the desired results, since these could be used as alternatives and be better for our health.

## 7 Conclusion

The increasing consumer demand for fresh and healthy juices, along with the FDA's concerns about the safety of low-acid juices (with pH levels of 5–6), has sparked the development of innovative and unconventional methods for preparing fruit and vegetable juices (FVJs). Non-thermal technologies (NTTs) have emerged as a promising solution, offering a range of benefits for the food and beverage industry. These cutting-edge methods not only preserve the natural freshness and nutritional value of juices but also boost their antioxidant capacity, safety, overall quality and acceptability of the juice. NTTs offer a game-changing solution for the industry, enabling the production of juices with minimal enzyme and microbial activity, and optimal nutritional and sensory properties. Nowadays, the majority of these methods are used on a small lab scale, leading to large-scale food industries. To unlock the full potential of non-thermal technologies, industries must first develop cost-effective machine designs and optimize process variables to minimize product damage. This crucial step will pave the way for widespread adoption and revolutionize the future of food processing. Moreover, combining multiple non-thermal technologies (NTTs) or integrating them with mild heating approaches yield excellent synergistic effects. Despite NTTs ability to eliminate foodborne microorganisms, improve nutrient quality and prolong shelf life, their commercial viability in the FVJs processing industry is limited due to their high fixed costs and scalability issues. To bridge this gap, further research is crucial to reduce costs and enhance the adaptability of NTTs for large-scale industrial applications, ultimately meeting the needs of both consumers and industries.

## CRediT authorship contribution statement

**Muhammad Umair:** Writing – original draft, Software, Methodology, Data curation, Conceptualization. **Muhammad Abid:** Writing – review & editing, Methodology, Formal analysis, Data curation, Conceptualization. **Mishal Mumraiz:** Writing – review & editing, Visualization, Formal analysis, Data curation, Conceptualization. **Saqib Jabbar:** Writing – review & editing, Validation, Resources, Methodology, Formal analysis, Data curation, Conceptualization. **Song Xun:** Writing – review & editing, Software, Investigation, Formal analysis, Data curation, Conceptualization. **Kashif Ameer:** Writing – review & editing, Writing – original draft, Validation, Project administration, Methodology, Formal analysis, Data curation. **Muhammad Shahid Riaz Rajoka:** Writing – review & editing, Resources, Investigation, Formal analysis, Data curation, Conceptualization. **He Zhendan:** Writing – review & editing, Writing – original draft, Supervision, Resources, Methodology, Formal analysis, Data curation. **Saqer S. Alotaibi:** Writing – review & editing, Software, Methodology, Funding acquisition, Data curation, Conceptualization. **Robert Mugabi:** Writing – review & editing, Validation, Investigation, Formal analysis. **Gulzar Ahmad Nayik:** Writing – review & editing, Supervision, Resources, Investigation, Funding acquisition, Conceptualization.

## Declaration of competing interest

The authors declare that they have no known competing financial interests or personal relationships that could have appeared to influence the work reported in this paper.
